# Reaching nearby sources: comparison between real and virtual sound and visual targets

**DOI:** 10.3389/fnins.2014.00269

**Published:** 2014-09-02

**Authors:** Gaëtan Parseihian, Christophe Jouffrais, Brian F. G. Katz

**Affiliations:** ^1^Laboratoire de Mécanique et d'Informatique pour les Sciences de l'Ingénieur, LIMSI - CNRS, Universite Paris SudOrsay, France; ^2^IRIT, CNRS, Université de ToulouseToulouse, France

**Keywords:** auditory localization, near-field pointing, nearby sound sources, virtual auditory display, spatial hearing, sound target, visual target

## Abstract

Sound localization studies over the past century have predominantly been concerned with directional accuracy for far-field sources. Few studies have examined the condition of near-field sources and distance perception. The current study concerns localization and pointing accuracy by examining source positions in the peripersonal space, specifically those associated with a typical tabletop surface. Accuracy is studied with respect to the reporting hand (dominant or secondary) for auditory sources. Results show no effect on the reporting hand with azimuthal errors increasing equally for the most extreme source positions. Distance errors show a consistent compression toward the center of the reporting area. A second evaluation is carried out comparing auditory and visual stimuli to examine any bias in reporting protocol or biomechanical difficulties. No common bias error was observed between auditory and visual stimuli indicating that reporting errors were not due to biomechanical limitations in the pointing task. A final evaluation compares real auditory sources and anechoic condition virtual sources created using binaural rendering. Results showed increased azimuthal errors, with virtual source positions being consistently overestimated to more lateral positions, while no significant distance perception was observed, indicating a deficiency in the binaural rendering condition relative to the real stimuli situation. Various potential reasons for this discrepancy are discussed with several proposals for improving distance perception in peripersonal virtual environments.

## 1. Introduction

The basic mechanisms of sound localization have been well studied in the last century (see Blauert, 1997). These studies have primarily examined azimuth and elevation localization accuracy using a variety of reporting techniques. Several studies have examined distance perception under a variety of acoustic conditions, though typically for frontally positioned sources in the far-field. Few studies have examined spatial hearing in the near-field and even fewer for positions significantly low in elevation.

For sources located in the near field, several studies (see Brungart and Rabinowitz, 1999; Shinn-Cunningham et al., 2000) have shown through analysis of proximal-region Head-Related Transfer Function (HRTF) measurements a dramatic increase in Interaural Level Difference (ILD) cues for sources within 1 m of a listener's head for positions away from the median plane. This increase is the consequence of two factors. First, due to head shadowing, the more proximate is the source from the head, the more high frequency attenuation is observed on the contra-lateral acoustic trajectory. Second, as acoustic waves follow an attenuation inverse-square law relationship between distance and intensity, the differences in path length between the two acoustic trajectories reaching each ear for near sources is proportionally bigger than for far sources. This leads to greater and more easily noticeable ILD. In contrast, the Interaural Time Delay (ITD) cue is roughly independent of distance in the proximal region. Although there is a slight increase of ITD for nearest distances, it occurs only near the lateral positions where the ITD is large and where listeners are relatively insensitive to ITD changes (see Hershkowitz and Durlach, 1969). Considering the spectral cues' variation in near field, Brungart and Rabinowitz (1999) have shown that the features of the HRTF that significantly change with elevation are not strongly dependent on the distance. However, as the source approaches the head, the angle of the source relative to the ear can differ from the angle of the source relative to the center of the head. This creates an acoustic parallax effect that laterally shifts some of the high-frequency features of the HRTF (see Brungart, 1999).

Only few studies have aimed to evaluate sound source localization performances in the near-field. Ashmead et al. (1990) evaluated the perception of the relative distances of frontal sources near one and two meters with only intensity cues in an anechoic room. They found a smallest noticeable change in distance of 5% (e.g., a distance of 5 cm at 1 m) whereas Strybel and Perrott (1984) found a change of 10% and Simpson and Stanton (1973) of 20%. Concerning the response methods for the localization of nearby object, Brungart et al. (2000) compared four response methods with visual targets and found a superiority of the direct pointing method over the other methods. With this method, the authors highlight an overall error of 7.6% in distance and of 5° in azimuth when subjects pointed toward visual targets. In an experiment performed to evaluate proximal-region localization performances, Brungart et al. (1999) found an increase in azimuth error as the sound approached the head, a distance independency of elevation performance, and a strong azimuth dependency of distance localization performances. This study was performed without amplitude-based distance cues using sources distributed from −40° to 60° in elevation, 15 to 100 cm in distance, and situated in the right hemisphere of the subject.

In Shinn-Cunningham et al. (2005), the authors analyzed the distortions of the spatial acoustic cues induced by the presence of reverberant energy. They measured Binaural Room Impulse Responses (BRIRs) on a KEMAR manikin for several nearby sound sources positions in a classroom. Their results highlighted a reduction of the ILD depending on acoustic properties of the environment as well as on the location of the listener in the environment. Furthermore, monaural spectral cues are less reliable in the ear farther from the sound source whereas ITD can still be recovered from the BRIRs. These systematics distortions are mostly prominent when the listener is oriented with one ear toward a wall. With a perceptual study on the effect of near field sound source spectrum on lateral localization in virtual reverberant simulation, Ihlefeld and Shinn-Cunningham (2011) showed a compression of the perceived angle toward the center for lateral sources (more than 45° from the median plane). This effect grows with increasing distance (as Direct/Reverberant ratio decreases) and it is greater for low-frequency sounds than for high-frequency sounds. Exploring the effect of simulated reverberant space on near field distance perception, Kopčo and Shinn-Cunningham (2011) showed lower performances for the evaluation of frontal sources than for lateral sources. They highlighted a high influence of sound spectrum on distance perception and explain it by assuming that near distances are evaluated using a fixed Direct/Reverberant mapping with distance that vary with frequencies.

Exploitation of the human capacities for spatial auditory perception often involves the creation of virtual auditory environments. The basis of this technique has been described in detail by Begault (1994) and Xie (2013). Such virtual reality simulations have been used in numerous studies, for example in the study of spatial cognition by Afonso et al. (2010), the treatment of phobias by Viaud-Delmon et al. (2008), the perception of architectural spaces by acoustic information by the blind by Picinali et al. (2014), interactive multidimensional data sonification and exploration by Férey et al. (2009), training systems to improve localization ability by Honda et al. (2007) and Parseihian and Katz (2012b), as well as in navigation systems for the blind by Wilson et al. (2007); Walker and Lindsay (2006). However, the vast majority of virtual auditory applications have focused on either far-field virtual sources, or virtual sources in the near horizontal plane and higher elevations. Very few studies have addressed very low elevations and proximal source positions.

In the context of the development of a specific integrated near- and far-field navigation and guidance system using spatial audio rendering (see Katz et al., 2012) this study concerns not only the accuracy in pointing to the direction of an auditory source (azimuth and elevation), but the accuracy in indicating the exact position of an anechoic auditory source. One situation of specific interest is the ability to locate an auditory source when positioned on a table-top type surface, which would be the position for which the user would be guided. This context considers both near and low elevation source positions.

The current study proposes an evaluation of basic auditory localization and pointing accuracy for sources low elevation in the peripersonal space. This condition examines an area rarely studied in previous literature. While not carried in anechoic conditions, the current study is performed in an acoustically damped room with very low reverberation. As such, the results can be compared to previous anechoic and non-anechoic condition studies, with the understanding that some minor room effect is present. Accuracy is evaluated as a function of pointing hand used, in an attempt to examine if there is any bias relative to hand dominance in the reporting task. This experiment explores localization and pointing accuracy for source positions spanning azimuths of ±120°.

A subsequent evaluation examines the potential of errors in position reporting due to biomechanical related effects rather than auditory perception limitations. A visual condition using the same test protocol serves as a control condition, with results showing that pointing accuracy is good and similar anywhere around the subject. To address the contextual situation, the subsequent study also includes a secondary preliminary investigation exploring how the peripersonal pointing accuracy depends on a virtual implementation of distance cues using an anechoic binaural simulation. This subsequent experiment explores localization and pointing accuracy over a reduced angular range, with source positions spanning azimuths of ±60°.

The following section provides an overview of the experimental design with each individual experiment being detailed in subsequent sections.

## 2. Reaching to sound sources

In order to investigate sound localization and pointing accuracy in the peripersonal space, two exploratory experiments have been designed and carried out. The first experiment evaluates general localization accuracy and specifically examines the effect of the pointing hand, dominant vs. secondary, for the pointing task. The second experiment compares the localization and pointing accuracy in peripersonal space for two additional stimulus types relative to the first experiment. Firstly, comparisons are made to visual stimuli, in an attempt to identify any common reporting errors due to difficulties relating to the pointing task. Secondly, the experimental platform is reproduced using a virtual audio display employing binaural synthesis, in an attempt to provide a benchmark for localization and pointing accuracy in the peripersonal space in a virtual environment.

All experiments were carried out in the same conditions, using the common protocol and experimental platform, in order to facilitate comparisons. Details of the protocol and platform are provided in the following section. Specific details associated with a given experiment are described in the subsequent sections along with the results of the two experiments.

### 2.1. Stimuli

A brief sound stimulus was used for the two experiments in order to prevent active localization related to dynamic cues during head movement. It consisted of a train of three, 40 ms Gaussian broadband noise bursts (50–20000 Hz) with 2 ms Hamming ramps at onset and offset and 30 ms of silence between each burst. This stimulus was chosen following (Macé et al., 2012), where the effect of repetition and duration of the burst on localization accuracy was analyzed for blind and sighted individuals. Their results showed an improvement in accuracy between three repeated 40 ms bursts and a single 200 ms burst. The overall level of the train was approximately 60 dBA, measured at the ear position.

### 2.2. Setup

**Figure 1:**
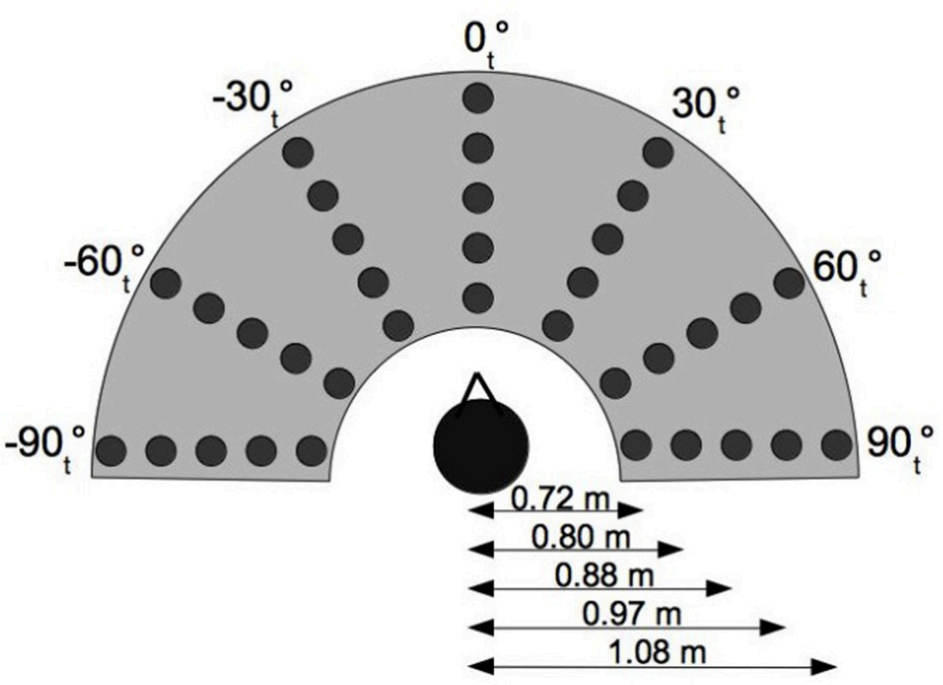
**Experimental setup**. θ_*t*_ indicates the source position in the subjects head coordinate system.

The experimental setup used for both experiments consists of a semicircle platform of 1 m radius. It contained 35 sound sources distributed on five semi-circular rows spaced by 13 cm (radii at: 33, 46, 59, 72, and 85 cm); each row contained seven sources spaced by 30° (Figure 1). For the real sound condition, the sources comprised 35 small loudspeakers (ref: CB990, 8 Ohms, 3 Watt) placed under an acoustically transparent grid. Acoustically absorbing foam covered the mounting board between loudspeakers. Subjects were seated on a swivel chair with their head placed over the center of the semi-circle and at a height of 65 cm. Each loudspeaker was oriented to the subject's head in order to avoid loudspeaker directivity variations. All sources were equalized to present the same spectral response (speakers responses were flattened with twelve cascaded biquad filters) and level calibrated at the center of the listening head position (± 1 dB SPL). The spectral equalization suppressed potential supplementary localization cues and learning effects for a given loudspeaker. The loudness equalization suppressed the distance attenuation intensity cue so as to avoid potential relative judgments and to place the listener in an unfamiliar condition (the subject doesn't know the source and its “natural” level) as in Brungart et al. (1999).

For the first experiment the subject's head was tracked with a 6-DoF position/orientation sensor (Optitrack motion capture system—precision: 0.2° in azimuth, 5 mm in distance) positioned on the top of the head, and hand position was tracked with a sensor positioned over the extremity of the hand (on the tip of the three middle fingers). The position of the hand was calculated relative to the 6-DoF head tracker shifted to the center of the head. For the second experiment, hand positions were measured with the Optitrack motion capture system and head orientation was monitored with a magnetic sensor [Flock of Bird, Ascension Technology—angular precision (yaw, pitch, roll): 0.5°].

The experimental setup was located in a dark and acoustically damped low reverberant space (reverberation time ≈300 ms in the mid frequency region) in order to avoid any visual or auditory cues from the experimental platform and surrounding environment.

## 3. Experiment 1: Pointing hand effect

The aim of experiment 1 was to measure pointing/reaching accuracy toward real sound sources. This accuracy was then evaluated as a function of the pointing hand and source location. In addition to source position, the effect of the reporting method, specifically dominant vs. non-dominant hand, was evaluated.

### 3.1. Materials and methods

#### 3.1.1. Subjects

A total of 15 adult subjects (3 women and 12 men, mean age = 28 years, *SD* = 6) served as paid volunteers; all were healthy. An audiogram was performed on each subject before the experiment to ensure that his or her audition was normal (defined as thresholds no greater than 15 dB hearing level in the range of 125–8000 Hz). All were naive regarding the purpose of the experiment and the sets of spatial locations selected for the experiment. All were self-reported right handed; no handedness measure was performed to establish their dominant hand. This study was performed in accordance with the ethical standards of the Declaration of Helsinki (revised Edinburgh 2000) and written informed consent was obtained from all subjects prior to the experiment (after receiving instructions about the experiment).

#### 3.1.2. Experimental procedure

The localization task consisted in reporting the perceived position of a brief static sound sample using a hand placing technique. Each subject was instructed to orient him- or her-self straight ahead and keep his/her head fixed, in a reference position at the center of the system, 0.65 m over the table, during the brief sound stimulus presentation. Before each trial, the subject's head position was automatically compared to the reference position and the subject was asked to correct the position if there was no concordance (± 5 cm for position and ± 3° for orientation). After presentation of the stimulus, the subject was instructed to place the tip of his/her hand on the table at the location of the perceived sound source and to validate the response with a MIDI button in front of the subject, placed near the center of the inner arc, using their other hand. In this manner, the subject was stationary during stimulus presentation, avoiding dynamic cues. The reported position was calculated between the initial head center position/orientation when the stimulus was played and the final extremity of the hand position when the listener validated the target. No feedback was provided regarding the actual target location.

Preliminary experiments using the semi-circle table showed that sources at the extreme azimuths posed problems as they were too close to the edge of the table which unintentionally provided subjects a tactile reference point. As such, the experimental protocol was modified to use only 25 sources (from −60°_*t*_ to 60°_*t*_ in Figure 1) with two subject orientations in order to cover a larger range of tested relative azimuths. For each hand, a total of 35 sources were tested with 7 different azimuths (−60°_*r*_, −30°_*r*_, 0°_*r*_, 30°_*r*_, 60°_*r*_, 90°_*r*_, and 120°_*r*_), where θ_*r*_ represents the source azimuth relative to the subject's head orientation and θ_*t*_ represents the source azimuth in the table reference frame. The experiment was realized in four phases:

- Subject faced the −60°_*t*_ line and reported 25 source locations (0°_*r*_, 30°_*r*_, 60°_*r*_, 90°_*r*_, and 120°_*r*_) using the 1st (dominant, right) hand.
- Subject faced the −60°_*t*_ line and reported 15 source locations (−60°_*r*_, −30°_*r*_, and 0°_*r*_) using the 2nd (non-dominant, left) hand.
- Subject faced the +60°_*t*_ line and reported 25 source locations (0°_*r*_, 30°_*r*_, 60°_*r*_, 90°_*r*_, and 120°_*r*_) using the 2nd (non-dominant, left) hand.
- Subject faced the +60°_*t*_ line and reported 15 source locations (−60°_*r*_, −30°_*r*_, and 0°_*r*_) using the 1st (dominant, right) hand.

All locations were repeated 5 times and randomly presented for each phase in five blocks of 25 locations (phases *a* and *c*) or 15 locations (phases *b* and *d*). A total of 400 locations were presented during the experiment and the total duration was around 90 min.

#### 3.1.3. Data analysis

Because of technical validation problems with several participants (some subjects had a tendency to validate the reported position before the end of their hand placement movement), all trials with reported positions significantly above the table's surface (>10 cm) have been removed from further analysis (0.68% of all the trials).

Accuracy was calculated by measuring the bias and dispersion between the sound source and reported position in head spherical coordinates (azimuth, elevation, and distance). Due to the platform's configuration, distance and elevation of the source relative to the head are interdependent. As such, results were analyzed across two components: azimuth and distance relative to the subject.

As source locations were calculated in head coordinates, initial distances of 0.33, 0.46, 0.59, 0.72, and 0.85 m from the center of the platform corresponded to actual distances of *d*_1_ = 0.729, *d*_2_ = 0.796, *d*_3_ = 0.885, *d*_4_ = 0.970, and *d*_5_ = 1.078 m from the center of the head (located 0.65 m above the platform).

Some front/back confusion errors were observed for rendered sources at lateral positions. These were identified according to the conventional definition of front/back confusion (proposed by Wightman and Kistler, 1989): if the angle between the target and the judged position is bigger than the angle between the target and the mirror of the judgment about the interaural axis, the judgment is considered as a confusion; combined with exclusion zone of Martin et al. (2001) (both the target and the judged position of the sound source do not fall within a narrow exclusion zone of ± 7.5° around 90° axis). Due to the occurrence of such confusions (8.3% of all the trials), the analysis were performed both on the azimuth with front/back confusions present and on the azimuth after correcting front/back confusions by mirroring the judgment across the interaural axis (“corrected azimuth”).

Statistical analyses were performed with repeated measurement analysis of variance (ANOVA) after verifying the datas distribution normality of unsigned azimuth error and signed distance error with Shapiro-Wilk tests on each hand, azimuth and distance conditions (only two conditions over fourteen were slightly skewed for the azimuth error). A Tukey *post-hoc* was used to assess differences between conditions.

### 3.2. Results

**Figure 2:**
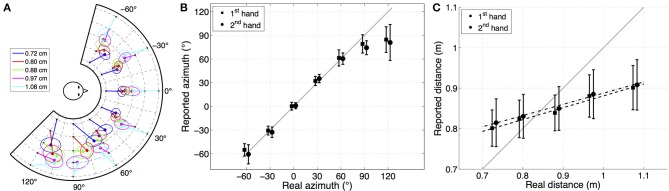
**(A)** (Color on-line) Mean of all subjects' reported location with 50% confidence ellipse linked to source location for the dominant hand condition. Front/back confusion corrected. Good directional pointing accuracy in the median plane, larger compression of reported distances in front than in side. **(B)** Mean of all subjects' reported azimuth as a function of the target azimuth for both hand conditions. Error bars show one standard deviation across the subjects. Gray line shows unity. For the sake of readability, results corresponding to the different hand conditions have been slightly horizontally shifted. This plot shows a good pointing accuracy on the frontal hemisphere and lower accuracy on the side. **(C)** Mean of all subjects' reported distance as a function of target distance with mean of linear regression slope for 1st and 2nd hand across all azimuths. Gray line shows unity. Error bars show one standard deviation across the subjects. Reported distance is linear but compressed.

Figure 2A presents the mean pointed position (with corrected azimuth) for the 1st and 2nd hand condition with the precision estimated by the 50% confidence ellipse linked to each position. For each target, 50% confidence ellipses were computed across all the subjects and all the conditions according to the method proposed by Murdoch and Chow (1996). The angle of the ellipse is determined by the covariance of the data and the magnitudes of the ellipse axes depend on the variance of the data. These plots highlight a compression of the reported distance dependent on the stimuli angle and a shift of the reported azimuth dependent on the stimuli angle and distance. For example, the azimuth error at 90° is larger for nearer sources and distance perception appears better at lateral angles. Azimuth and distance errors have been analyzed as a function of reporting hand, stimuli azimuth, and stimuli distance.

#### 3.2.1. Azimuth error

Figure 2B represents the reported azimuth (without front/back confusion correction) as a function of stimuli azimuth. Unsigned azimuth error and front/back confusion rates as a function of azimuth are presented in Table 1. These results highlight good pointing accuracy with low variability in the frontal direction (−30° ≤ θ_*r*_ ≤ 30°) with a mean unsigned error of 6.7°, and lower accuracy with greater variability toward the sides with a mean error of 17.8°. Lateral locations were underestimated. Front/back confusion analysis (Table 1) shows high levels of confusions from back to front at 120° and some confusions from front to back at −60° and 60°. The azimuth error at 120° when correcting front/back confusions is 15.9° against 39.2° without corrections. Surprisingly, one can observe some front/back confusions at 0° and 30° although the subjects were aware of the platform geometry. The standard deviation of azimuth error (around 15°) shows the high inter-subject variability. The mean of the standard deviation of azimuth error over subjects (7.9 ± 3.2°) shows a lower intra-subject variability.

**Table 1:** **Mean of absolute azimuth and corrected azimuth error in degree (standard deviations in parenthesis) and front/back confusion rate as a function of stimuli azimuth**.

	**Azimuth**	**−60**°	**−30**°	**0**°	**30**°	**60**°	**90**°	**120**°	**Total**
Azimuth	1st hand	9.3 (7.8)	6.7 (5.4)	6.5 (7.8)	7.1 (5.8)	11.1 (8.9)	15.2 (12.4)	36.1 (12.4)	12.3 (14.6)
Error	2nd hand	11.9 (9.7)	6.9 (5.9)	6.1 (7.4)	8.2 (7.3)	9.2 (8.0)	17.7 (12.0)	42.0 (23.5)	13.6 (16.2)
Corrected	1st hand	9.3 (7.8)	6.7 (5.4)	6.4 (5.9)	7.1 (5.8)	10.7 (8.1)	15.2 (12.4)	15.9 (11.8)	9.7 (9.1)
azim. error	2nd hand	11.3 (8.7)	6.9 (5.9)	6.0 (5.4)	8.1 (7.2)	9.2 (7.9)	17.7 (12.0)	11.8 (8.8)	9.6 (8.7)
F/B	1st hand	0.0	0.0	0.1	0.0	3.5	0.0	56.5	7.5
Conf. (%)	2nd hand	4.3	0.0	0.1	0.3	0.8	0.0	66.3	9.1

Overall performance shows similar errors in the median plan and greater differences on the side, but with a slight difference when using the dominant (1st) or non-dominant (2nd) hand. A repeated measure ANOVA performed on front/back confusion rate with pointing hand as a factor shows no significant differences between the two conditions [*F*_(1, 14)_ = 2.53, *p* = 0.13]. A repeated measure 3-factor ANOVA (Hand*Azimuth*Distance) performed on the absolute corrected azimuth error highlights a significant effect of azimuth [*F*_(6, 84)_ = 19.14, *p* < 10^−5^] and distance [*F*_(4, 56)_ = 6.63, *p* < 0.001] but no effect of hand reporting condition [*F*_(1, 14)_ = 0.01, *p* = 0.91]. The *post-hoc* analysis performed on the azimuth indicates significant differences in performance between central positions (−30°, 0°, and 30°), lateral positions (−60° and 60°), and extreme positions (90° and 120°). The *post-hoc* analysis performed on distance highlights significantly poorer azimuth estimation for the nearest positions (*d* = 0.33 m). Interaction analysis shows an effect of Hand*Azimuth [*F*_(6, 84)_ = 4.59, *p* < 0.001] with significant differences in azimuth performances for the 1st and 2nd hand condition at 120°; no interaction effect of Hand*Distance [*F*_(4, 56)_ = 0.35, *p* = 0.84]; and an interaction effect of Azimuth*Distance [*F*_(24, 336)_ = 7.43, *p* < 10^−5^].

#### 3.2.2. Distance error

Figure 2C shows the average mean reported source distance as a function of sound source distance. This figure highlights a compressed but still linear perception of distance in the range of the tested region. The mean distance error across subjects, and the slope of the regression line and goodness-of-fit criteria *r*^2^ calculated over the five trials for each azimuth and hand condition are shown in Table 2.

**Table 2:** **Mean of absolute distance error (standard deviations in parenthesis), slope of the regression line and goodness-of-fit criteria *r*^2^ for each azimuth and hand condition**.

	**Azimuth**	**−60**°	**−30**°	**0**°	**30**°	**60**°	**90**°	**120**°	**Total**
Absolute distance	1st hand	9.5 (7.6)	9.9 (7.7)	10.7 (8.9)	10.5 (8.4)	8.7 (6.3)	8.6 (6.4)	10.0 (7.4)	9.8 (7.8)
error (cm)	2nd hand	9.8 (7.8)	10.6 (8.5)	10.5 (8.5)	10.1 (8.1)	9.0 (7.1)	9.4 (7.2)	12.1 (9.2)	10.3 (8.2)
Regression	1st hand	0.31 (0.17)	0.29 (0.14)	0.21 (0.16)	0.25 (0.12)	0.38 (0.12)	0.40 (0.17)	0.30 (0.19)	0.31 (0.15)
slope	2nd hand	0.34 (0.14)	0.24 (0.12)	0.24 (0.15)	0.26 (0.15)	0.35 (0.14)	0.34 (0.14)	0.22 (0.21)	0.28 (0.15)
Goodness-	1st hand	0.53	0.46	0.22	0.38	0.60	0.51	0.32	0.43
of-fit *r*^2^	hand	0.55	0.40	0.31	0.41	0.52	0.48	0.23	0.41

These results highlight difficulty regarding distance perception and a tendency to overestimate sound distance for the two nearest distances and to underestimate it for the others. Global error is ≈10 cm which equated to 11% relative error. This error is lower at the sides (9.2 cm) than toward the front (10.4 cm). Although distance perception was compressed (with a mean regression slope of 0.30 ± 0.11), Figure 2C shows a quasi linear perception of distance in the range of the tested region (36 cm). The comparison of regression slope across stimuli angles (Table 2) highlights better distance perception to the side (at −60°, 60°, and 90°) than toward the front. Standard deviation of the distance error (around 8 cm) indicates high inter-subject variability. The mean of the standard deviation of the distance error across subjects (7.6 ± 1.0 cm) also indicates intra-subject variability. Regarding the mean of the regression slope across subjects (*m* = 0.29 ± 0.11), inter-subject variability is quite large (the subject with lowest distance perception obtaining a mean slope of 0.12 ± 0.18 and the subject with highest distance perception a mean slope of 0.52 ± 0.11).

Performance analysis as a function of reporting hand condition showed few differences between 1st and 2nd hand. In both cases, distance perception was virtually the same, however, with higher variability in the 2nd hand condition. A repeated measure 3-factor ANOVA (Hand*Azimuth*Distance) performed on signed distance error highlights a significant effect of azimuth [*F*_(6, 84)_ = 70.09, *p* < 10^−5^], a significant effect of distance [*F*_(4, 56)_ = 572.78, *p* < 10^−5^] and no effect of hand [*F*_(1, 14)_ = 0.88, *p* = 0.36]. The *post-hoc* analysis performed on the azimuth shows significant differences in distance evaluation between 60° and 90° positions (which lead to the best distance evaluation) and the others positions. The *post-hoc* analysis performed on distance highlights significant differences between all distances positions, with over estimation of the distance for nearest positions (*d*_1_ and *d*_2_) and under estimation for the others (*d*_3_, *d*_4_, and *d*_5_). Interaction analysis shows an effect of Azimuth*Distance [*F*_(24, 336)_ = 4.47, *p* < 10^−5^]; no effect of Hand*Azimuth [*F*_(6, 84)_ = 1.62, *p* = 0.15] and no effect of Hand*Distance [*F*_(4, 56)_ = 1.82, *p* = 0.14].

Regression slopes are virtually equal in the two conditions (0.30 ± 0.11 for 1st hand and 0.28 ± 0.11 for 2nd hand) as well as the goodness of fit. A 2-factor ANOVA (Hand*Azimuth) performed on the regression slope showed no effect of the reporting hand [*F*_(1, 14)_ = 3.36, *p* = 0.09] and an effect of the azimuth [*F*_(6, 84)_ = 4.47, *p* < 0.001]. The *post-hoc* test revealed a significant difference between regression slopes calculated for 0° azimuths and those calculated for 60° and 90° azimuths. No Hand*Azimuth interaction was observed.

Distance accuracy in the studied zone was poorer than azimuth accuracy. Although distance error clearly depends on the distance of the stimuli (linear regression), it is compressed toward the center of the reporting area.

### 3.3. Discussion

The results of this experiment show a large variability between subjects. Despite this disparity, the results highlight the capacity of listeners to perceive and report a sound target within a general error of ≈ 13°, and an error of ≈ 6–7° in the frontal zone. In this zone, distance perception is poorer and compressed to the middle of the platform. Although distance perception was almost linear in the range of the tested region, the low value of the regression slope (around 0.3) highlights the difficulty in perceiving and reporting target stimuli using the sound cues provided.

The poor performance for distance perception can be explained in several ways. First, the small range variation of distances (from 0.72 to 1.08 m, total variation 0.36 m) is an important limitation factor. Second, the normalization of the stimulus amplitude to eliminate global distance attenuation cues and relative level differences makes distance judgments more difficult. Third, the suppression of the reverberant field with absorbent material reduces potential distance cues due to binaural variations and spatially coherent reflection information.

Since the setup of this experiment differs from previous studies, precise comparison is difficult. It is however possible to make comparisons with Brungart et al. (1999) (carried out in an anechoic room), considering their results for frontal and lateral zones with elevations below −20° and distances between 0.5 and 1.0 m. For azimuth perception, Brungart et al. (1999) reported a lateral error of 13.4° (between 60° and 120°) and a frontal error of 16.1° (between −60° and 60°). Results of our study, with errors of 18° for lateral angle and 7° for the frontal zone, show an opposite trend to their results. First, this difference can be explained by the front/back confusion suppression applied by Brungart et al. (1999). Results are more similar with suppression of front/back confusions in our results (lateral error ≈ 13°). Despite this, the difference in azimuth error in the frontal zone is surprising. In our study, subjects were more precise in the frontal zone, which is coherent with classical localization results summarized by Blauert (1997) for greater distances. One explanation could be the difference in reporting technique; reported locations were calculated relative to a position sensor mounted on the end of a 20 cm wooden wand in Brungart's experiment and directly to the hand in the present experiment. For distance perception, studies from Brungart et al. (1999) and Kopčo and Shinn-Cunningham (2011) reported more accurate results than the current work, with regression slopes around 0.90 in the lateral zone and around 0.70 in the frontal zone (compared to 0.34 and 0.25 respectively in the current study). Although the three studies show the same tendency of improved distance perception for lateral positions, the distance perception in the current study is significantly worse. One can note the different range of distances value used in these studies (from 0.7 to 1.1 m in our study, from 0.1 to 1.0 m in Brungart's study, and from 0.15 to 1.7 m in Kopčo and Shinn-Cunningham, 2011). One major difference in test conditions was that elevation angles varied from approximately −65° to −37° in the current study while they varied from approximately −40° to −20° in Brungart et al. (1999) and were equal to zero in Kopčo and Shinn-Cunningham (2011). Where as Brungart's experiment took place in anechoic condition (with ILD as the main distance cue), and Kopčo and Shinn-Cunningham (2011)'s experiment in low reverberant conditions (TR ≈ 600 ms, with ILD and D/R as the main distance cues), the present experiment took place in an acoustically damped low reverberant space (TR ≈ 300 ms), where it can be assumed that listeners used both ILD and few near-ear D/R changes with distance, but also HRTF changes correlated to elevation's variations. Thus, the low distance perception in this study might be principally due to the small range of distances rather than the lack of distance cues. Future experiments in anechoic field are necessary to evaluate the influence of elevation cues in this type of situation.

Some bias for the nearest positions to the side may be linked to biomechanical limitations. Effectively, it is difficult to correctly place the hand near the body for lateral positions (especially at azimuth from 60° to 120°) and this may influence results by shifting the pointed position toward the center. However, similar compression and shift effects can be found in the results obtained by Soechting and Flanders (1989) with a pointing bias toward the remembered position of a short visual stimulus. In their experiment, results highlighted a slight compression of perceived distances when the platform was 40 cm below the head, and a slight shift toward the center for lateral targets (at −45° and −45°). Instead of arguing for biomechanical limitations, they showed that errors in pointing to remembered targets were due to approximations in sensorimotor transformations between extrinsic (target location in space) and intrinsic (limb orientation) reference frames (see Soechting and Flanders, 1989). This question is further addressed in experiment 2 which considers stimuli of different modalities, in order to identify common pointing task errors separate from the perceptual nature of the stimuli.

In summary, results highlight similar accuracy for pointing task toward sound sources in the frontal space with dominant and non-dominant hand. Common results as a function of hand choice allow for the elimination of reporting hand consideration in future experiments, offering greater flexibility in task design and reporting protocol for the participants.

## 4. Experiment 2: Real sound, virtual sound, and visual targets

The aim of experiment 2 was two-fold. In order to address questions concerning observed localization and pointing errors in certain regions as their cause being attributed to either perceptual or biomechanical limitations a visual stimulus was included, as a contrast to the auditory stimulus of the previous experiment. Any observed bias errors in both the auditory and visual conditions could indicate a common origin, such as biomechanical limitations in reporting accuracy to certain positions.

In order to address the applied context of using virtual or augmented audio reality for creating sound objects in the peripersonal space, and to identify possible limitations of current implementations of binaural rendering technology, a virtual audio stimulus was also included.

Due to the additional number of stimulus conditions, the range of stimulus positions was reduced to ±60°.

### 4.1. Material and methods

#### 4.1.1. Subjects

A total of 20 adult subjects (3 women and 17 men, mean age = 26 years, *SD* = 4), different from the first study, served as paid volunteers. An audiogram was performed on each subject before the experiment to ensure that their audition was normal (defined as thresholds no greater than 15 dB hearing level in the range of 125–8000 Hz). All were naive regarding the purpose of the experiment and the sets of spatial positions selected for the experiment. This study was performed in accordance with the ethical standards of the Declaration of Helsinki (revised Edinburgh 2000) and written informed consent was obtained from all subjects prior to the experiment (after receiving instructions about the experiment).

#### 4.1.2. Experimental procedure

As with the first experiment, the task consisted in reporting the perceived location of a static remembered target using a hand placement technique validated by a MIDI button. The experimental procedure was the identical to the first experiment (see Section 3.1.2) except that the reference position was 0.60 m above the table surface (0.05 m lower than the first experiment due to tracking instabilities). This experiment was divided into three blocks of 100 trials, each block lasting approximately 15 min and corresponding to a different condition (real sound, virtual sound, and visual target). For each condition, 25 locations were tested with 5 different azimuths (−60°, −30°, 0°, 30°, and 60°) and 5 different distances (33, 46, 59, 72, and 85 cm). All locations were repeated 4 times. Each condition was divided in 4 blocks (for the four repetitions) with a pseudo-random order for the locations. The stimuli used for the three conditions were:

- *Visual*: single 200 ms flash of a white disc (same total duration as the two sound conditions) having a 1 cm diameter, projected on the table covered by a black cloth using an overhead video projector;
- *Real sound*: three repeated 40 ms bursts rendered over loudspeaker's table as in experiment 1;
- *Virtual sound*: three repeated 40 ms bursts rendered over stereo open ear headphone (model Sennheiser HD570) spatialized using a non-individual HRTF set measured on a KEMAR mannequin (described in Section 4.1.3).

All stimuli were presented in the peripersonal space and were off before the beginning of the reporting movement.

The order of the two sound conditions was counterbalanced in order to suppress any potential learning effect. The visual condition was always at the end of the experiment so as to not influence the subject with the location of the sound sources.

#### 4.1.3. KEMAR HRTF

The HRTF of a KEMAR mannequin was measured for the purpose of this experiment. The measurement was performed in an anechoic room (see LISTEN, 2004 for room details). The mannequin was equipped with a pair of omnidirectional in-ear microphones (DPA 4060) according to a blocked meatus protocol. The mannequin was fixed to a metal support that followed the axis of a motorized turntable (B&K 9640), which allowed variation of its orientation within the horizontal plane. The interaural axis of the KEMAR dummy head was centered (at 1.9 m from the loudspeaker) using a set of three coincident laser beams. The axis of the turntable coincided with a line extending through the center of the dummy head, therefore minimizing displacements during rotations of the turntable. The measurement set was obtained using the sweep-sine excitation technique at a sample rate of 44.1 kHz (RME Fireface 800 audio interface). The free-field HRTF was obtained through normalization (direct deconvolution through division in the complex frequency domain) by the free-field system response without the KEMAR present. The resulting HRIR was windowed (rectangular) to a length of 256 samples. The window was positioned to include 20 samples before the first peak as evaluated over all positions. In order to render all the sound source positions, it was necessary to measure the HRTF over the entire sphere. The set used contained measurement from −90° to 90° in elevation in steps of 5°, and from −180° to 180° in azimuth in steps of 5°.

The HRTF was decomposed into spectral component (representing spectral cues) and pure delay (representing ITD cues). A spatial interpolation of the spectral component was realized (see Aussal et al., 2013). The spatialization engine used a hybrid HRTF, where the modeled individual interaural time difference (ITD) based on head and shoulder circumference (see Aussal et al., 2012) was combined with the KEMAR spectral component. Binaural sound sources were rendered using a real-time spatialization engine based on full-phase HRIR convolution. ILD cues were modified to account for contralateral level difference for near distances using a spherical head model and a parallax effect correction was implemented for distances inferior to 1 m (HRIR were selected taking into account the angle of the source relative to the ear rather than the angle relative to the head center) (see Katz et al., 2011, 2012).

Thus, in the *virtual sound* condition, the available distance cues consisted of ILD variations as well as localization spectral cues associated with the corrected HRTF angles position. No near field correction was made for ITD variations with distance. Furthermore, an additional distance cue consisted in the spectral variations corresponding to the elevation changes linked to the distance due to the configuration of the table top setup. No additional propagation paths or reflections were simulated.

In order to improve reporting performance of the binaural rendering using non-individual HRTF, three preliminary adaptation sessions of 12 min were conducted according to the method proposed by Parseihian and Katz (2012b). Briefly, this method consists of a training game allowing the subject to perform a rapid exploration of the spatial map of the virtual rendering by an auditory-kinesthetic closed-loop. These training sessions were performed 3 days in a row, 12 min per day, the last session being immediately prior to the main experiment.

#### 4.1.4. Data analysis

Analysis of results was performed following the same parameters as the first experiment: azimuth and distance relative to the subject. With subject's head located 0.60 m over the table, the sources were at distances of 0.685, 0.756, 0.842, 0.937, and 1.040 m from the subject's head center.

No front/back confusions (observed as pointing to the back) were observed. However, during the debriefing, some subjects reported having heard sources behind them or inside their head and pointed toward the table edge. As it is difficult to detect these pointing instances as front/back confusions, we looked for outliers in the dataset. First, a total of 48 trials with reported positions lying outside the table were preliminarily removed from the data (0.80% of all the trials; 0.00% of *real sound* condition trials, 1.55% of *virtual sound* condition trials, and 0.85% of *visual* condition trials.) Second, trials with signed azimuth error or signed distance error exceeding a fixed limit were removed. The upper limit was defined as *Q*_3_ + 1.5 × (*Q*_3_ − *Q*_1_) and the lower limit as *Q*_1_ − 1.5 × (*Q*_3_ − *Q*_1_) with *Q*_1_ and *Q*_3_ respectively the first and the third data quartile. Outside these limits the reported locations were tagged as outliers. A total of 244 trials were removed from the data (4.11% of all the trials; 6.45% of *real sound* condition trials, 2.14% of *virtual sound* condition trials, and 4.45% of *visual* condition trials.)

Statistical analyses were performed with repeated measurement analysis of variance (ANOVA) after verifying the datas distribution normality of unsigned azimuth error and signed distance error with Shapiro-Wilk tests on each hand, azimuth and distance conditions. A Tukey *post-hoc* was used to assess differences between conditions.

### 4.2. Results

**Figure 3:**
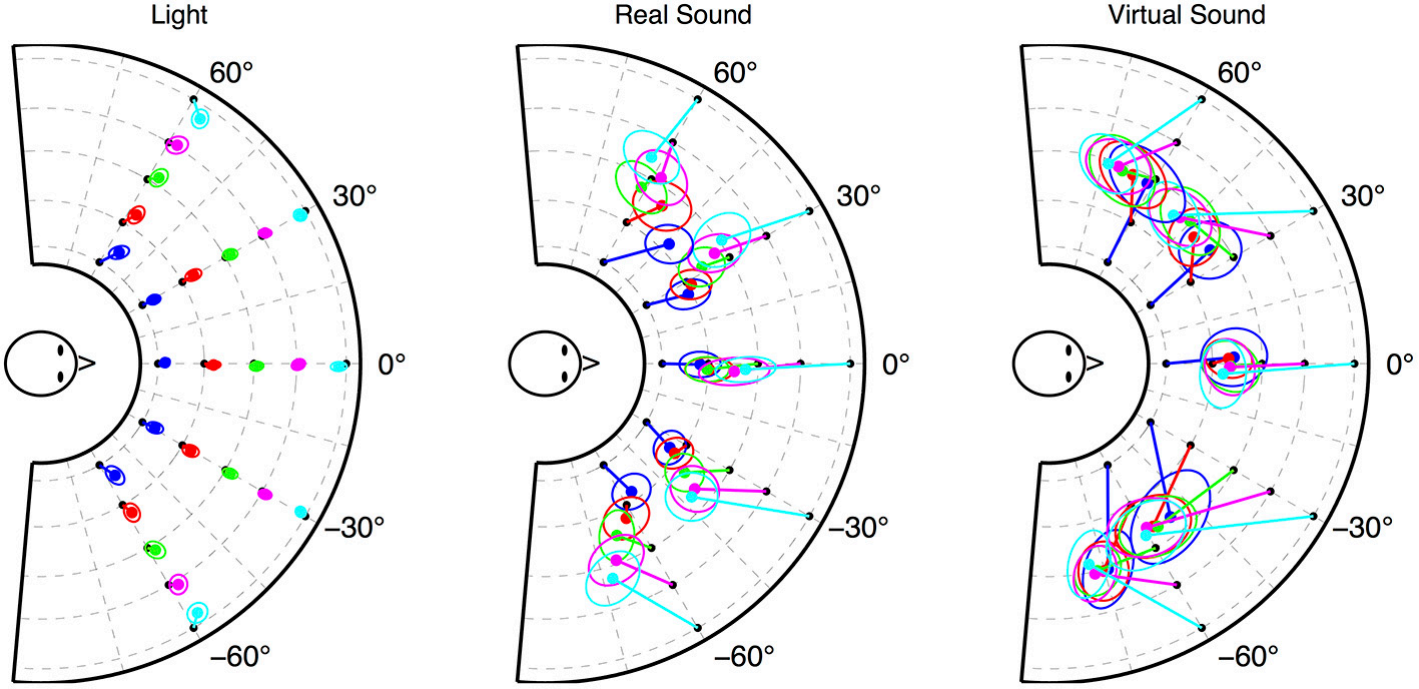
**Mean of all subjects' reported location with 50% confidence ellipse linked to source location for each rendering condition: *visual* (left), *real sound* (center), and *virtual sound* (right)**.

The mean reported positions linked to target locations for each rendering condition are presented in Figure 3 with 50% confidence ellipse. These plots allow one to evaluate the error bias across the three conditions. For *visual* sources, lateral localization accuracy is quite good while the nearest distances are overestimated. For *real sound* sources, the reported distance is compressed and a lateral shift appears mostly at −60° and 60°. For *virtual sound* sources, all lateral sources are shifted toward the sides and there is no apparent distance perception. In the following sections these results are analyzed in terms of azimuth and distance bias and dispersion.

#### 4.2.1. Azimuth error

**Figure 4:**
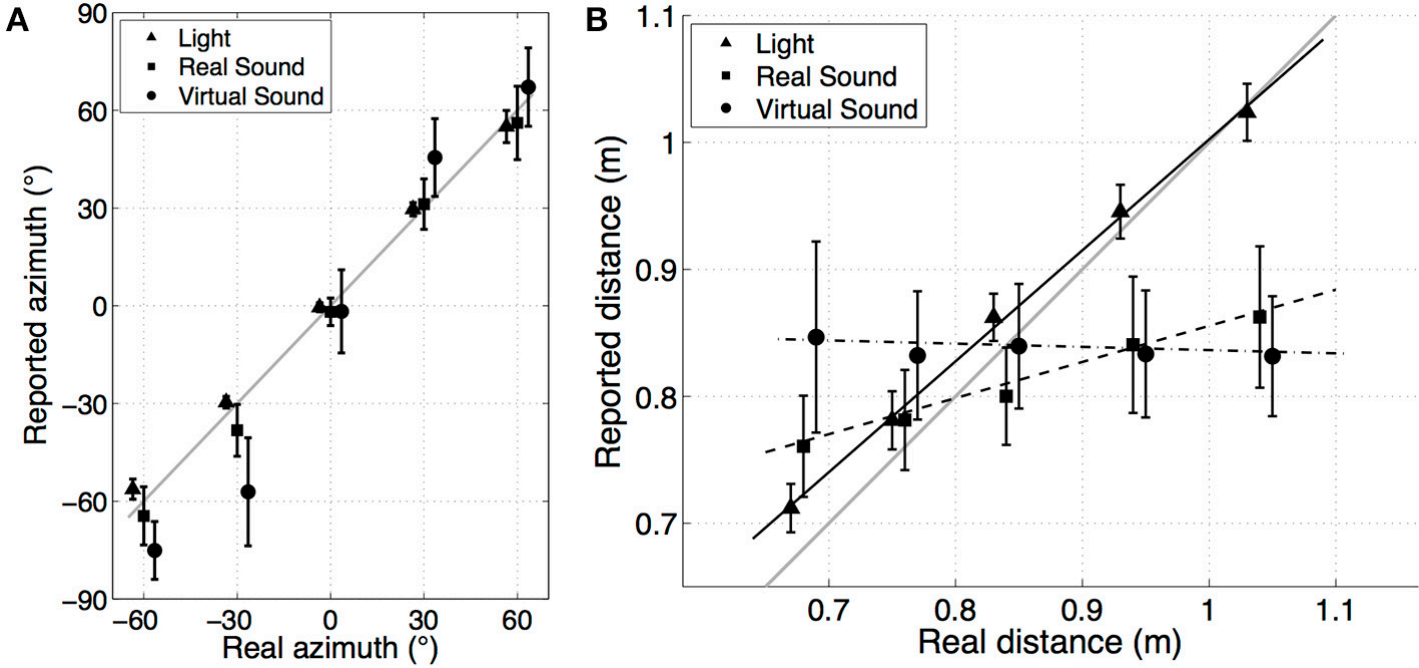
**(A)** Mean of all subjects' reported azimuth as a function of stimuli azimuth for each rendering condition: *visual* (▴), *real sound* (◾), and *virtual sound* (•). Error bars show one standard deviation across the subjects. Gray line shows unity. **(B)** Mean of all subjects' reported distance as a function of stimuli distance for each rendering condition: *visual* (▴), *real sound* (◾), and *virtual sound* (•). Error bars show one standard deviation across the subjects for each condition. Solid gray lines show linear regression curves for each modality. Gray line shows unity.

Figure 4A presents the mean and standard deviation of reported azimuth as a function of stimuli azimuth. The mean and standard deviation of the unsigned azimuth error are presented in Table 3. First, the *visual* condition shows good estimation of azimuth, with a low variability (mean error of 2.79° ± 4.51°). For frontal locations, the mean unsigned error is 1.61° ± 1.27°. This error increases with azimuth to 2° for ±30° locations and to 4° for ±60° locations, as does the dispersion. It can be noticed that the lateral error corresponds to a slight underestimation of the azimuth. Second, results for *real sound* condition are similar to the first experiment's results. They highlight good accuracy at 0° with a mean error of 5.7°, and lower accuracy at the sides. Third, *virtual sound* condition showed lower performance results in terms of azimuth estimation. The mean absolute error at 0° is 10.79° ± 10.03°. The ±30° and ±60° locations are shifted by approximately 15° to the side (except −30° locations which are reported at −60°).

**Table 3:** **Mean absolute azimuth error in degree (standard deviations in parenthesis) for each rendering condition as a function of stimuli azimuth**.

**Condition**	**−60**°	**−30**°	**0**°	**30**°	**60**°	**Total**
Visual	3.85 (5.84)	1.94 (2.97)	1.61 (1.27)	1.99 (2.56)	4.51 (6.56)	2.79 (4.51)
Real sound	11.08 (8.15)	10.66 (8.22)	5.70 (4.61)	7.08 (5.81)	11.30 (8.27)	9.18 (7.54)
Virtual sound	16.75 (9.04)	28.17 (17.43)	10.79 (10.03)	16.83 (13.78)	14.44 (9.97)	17.48 (13.76)

A repeated measures 3-factor ANOVA (Condition*Azimuth*Distance) was performed on the mean absolute azimuth error of each subject, highlighting a significant effect of condition [*F*_(2, 38)_ = 61.75; *p* < 10^−5^]. The *post-hoc* test revealed a significant difference between each condition. Azimuth errors are significantly lower for the *visual* condition compared to the sound conditions and are significantly greater for *virtual sound* condition compared to the two others. The interaction analysis showed a significant effect of Condition*Azimuth [*F*_(8, 152)_ = 15.62; *p* < 10^−5^] and of Condition*Distance [*F*_(8, 152)_ = 9.71; *p* < 10^−5^]. The *post-hoc* test on Condition*Azimuth highlights no effect of the azimuth on the *visual* condition, but it shows a significant difference of performance between 0° and lateral positions (−60° and 60°) for *real sound* condition and significant differences between 0° and (−60°, −30°, 30°) and between −30° and other angles for the *virtual sound* condition. For these two conditions the azimuth errors are significantly greater for lateral positions as compared to frontal positions. The *post-hoc* test on Condition*Distance highlights a significant difference on azimuth estimations between the nearest distance (*d*_1_ and *d*_2_) and the other distances (*d*_3_, *d*_4_, and *d*_5_). In this condition, the azimuth is better estimated for nearer distances.

#### 4.2.2. Distance error

Figure 4B shows the average mean response of reported distance as a function of stimuli distance for the three conditions. This figure highlights the large differences between the rendering conditions: the *visual* condition shows good and linear perception of distance, the *real sound* condition shows similar results as in the first experiment (e.g., compressed but linear perception of the distance in the range of the tested region), and finally there is no apparent distance perception in the *virtual sound* condition. A linear regression analysis was performed on these results. The mean of the linear regression line across the subjects for each rendering condition is shown in Figure 4B. The mean distance error across subjects, slope of the regression line, and goodness-of-fit criteria *r*^2^ calculated over the four trials for each azimuth and rendering condition are shown in Table 4. The overall mean results represent the mean of results for each subject when considering the entire data set (mean of subject's regression slopes calculated with all the data from one condition, without considering target azimuth).

**Table 4:** **Mean absolute distance error (standard deviations in parenthesis), slope of the regression line, and goodness-of-fit criteria *r*^2^ for each azimuth and rendering conditions**.

	**Azimuth**	**−60**°	**−30**°	**0**°	**30**°	**60**°	**Total**
Absolute	Visual	2.91(2.99)	2.17 (2.14)	1.88 (1.85)	2.22 (2.12)	3.12 (3.06)	2.47 (2.53)
distance	Real sound	8.68 (6.63)	9.88 (8.15)	10.82 (8.38)	9.50 (7.76)	9.05 (6.27)	9.58 (7.50)
error (cm)	Virtual sound	12.86 (9.53)	12.89 (9.39)	13.20 (9.46)	12.33 (8.82)	12.75 (8.64)	12.80 (9.16)
	Visual	0.88 (0.09)	0.91 (0.06)	0.93 (0.06)	0.91 (0.05)	0.85 (0.08)	0.89 (0.06)
Regression	Real sound	0.34 (0.18)	0.28 (0.16)	0.25 (0.21)	0.31 (0.19)	0.30 (0.16)	0.30 (0.18)
slope	Virtual sound	−0.05 (0.19)	−0.02 (0.22)	−0.03 (0.16)	0.00 (0.10)	−0.02 (0.24)	−0.02 (0.18)
	Visual	0.98	0.99	0.99	0.99	0.97	0.98
Goodness-	Real sound	0.52	0.46	0.31	0.46	0.44	0.44
of-fit *r*^2^	Virtual sound	0.10	0.10	0.09	0.05	0.18	0.11

First, the *real sound* condition results are similar to the first experiment with a mean absolute distance error of 9.6 ± 7.5 cm, and a mean regression slope of 0.30 ± 0.18. The evolution of the distance error as a function of stimuli angle is also as in the first experiment: distance perception was better for lateral angles than in the frontal space. Second, in the *virtual sound* condition, distance perception seems non-existent. The mean distance error is 12.80 cm and the variability covers a large part of the table with a standard deviation of 9.16 cm. The regression slope is practically zero (−0.02 ± 0.27) and the goodness-of-fit of 0.11 ± 0.11 shows that *virtual sound* distance perception cannot be considered as linear for each subject. Analyzing the regression slope as a function of subject shows that 11 subjects (out of 20) obtained a positive regression slope and only two subjects obtained a regression slope superior to 0.1. Third, *visual* condition shows good perception of distance with an absolute error of 2.5 ± 2.5 cm, a regression slope of 0.89 ± 0.06 and a goodness-of-fit of 0.98. Distance error analysis as a function of the target angle highlights a better distance perception in the frontal zone (mean distance error at 0° was 1.88 ± 1.185 cm) than in lateral zones (mean distance error at ±60° was 3.01 ± 3.02 cm).

A repeated measures 3-factor ANOVA (Condition*Azimuth*Distance) performed on the mean signed distance error shows a significant effect of the rendering condition [*F*_(2, 38)_ = 16.6; *p* < 10^−5^]. The *post-hoc* test revealed a significant difference between each condition with better performances obtained with *visual* condition and worst performances obtained with *virtual sound* condition. The analysis of Condition*Azimuth interaction [*F*_(8, 152)_ = 8.95; *p* = 0.005] shows a significant effect of the azimuth on the distance error in *real sound* condition between 0° and −60° and 60° and in *virtual sound* condition between 0° and the others angles positions. The analysis of Condition*Distance interaction [*F*_(8, 152)_ = 178.72; *p* < 10^−5^] highlights significant differences in distance error between furthest distance location (*d*_4_ and *d*_5_) and middle distance locations (*d*_2_, and *d*_3_) for the *real sound* condition and between middle distance location (*d*_3_) and extreme distance locations (*d*_1_, *d*_4_, and *d*_5_) for the *virtual sound* condition. A 2-factor ANOVA (Condition*Azimuth) performed on the regression slopes calculated for each subject shows a significant effect of the rendering condition [*F*_(2, 38)_ = 286.43; *p* < 10^−5^] (with each condition significantly different from the others) and no observed effect of azimuth and no interaction effect of Condition*Azimuth.

### 4.3. Discussion

The results of experiment 2 show a large inter-subject variability, as was observed in experiment 1, which is condition dependent (the highest inter-subject variability was observed for *virtual sound* condition whereas the lowest was observed for the *visual* condition). The results also highlight large differences in localization/pointing accuracy toward *light*, *real sound*, and *virtual sound* targets both in azimuth and distance.

Results for *real sound* condition show the same performances in azimuth and distance as for experiment 1 in the studied area. Distance perception is almost linear in the range of the tested region but largely compressed to the middle of the platform (regression slope of 0.3). Azimuth perception is better in the frontal zone (|*azimuth*| ≤ 30°) than toward the sides (|*azimuth*| = 60°). As seen in experiment 1, some localization biases are observed on the sides. For example, at 60° the azimuth of nearest source positions are underestimated whereas at −60° the azimuth of the farthest source positions is overestimated.

Results of the *virtual sound* condition are significantly poorer than those of the *real sound* condition. Directional pointing is shifted to the side for positions outside the median plane and there is no apparent distance perception (regression slope of 0). This could be attributed to the use non-individual HRTFs, despite the training period. Azimuth distortion errors could be attributed to the ITD individualization model employed and associated errors in the tested region, which exceeds the initial bounds of the developed method. This shift in azimuth perception is common with virtual auditory display and is smaller than the shift observed for example by Boyer et al. (2013) citing an overall azimuth error of 25°, as compared to 17.5° in the present study. However, these results show an opposite trend to the results of Ihlefeld and Shinn-Cunningham (2011), who observed a bias toward the median plane for perceived lateral angle sources more than 45° from the median plane. This difference might be explained by the presence of a reverberant field in the non-individualized BRIR used in their study (since this shift toward the center seems to be linked to the D/R ratio). In addition, the binaural rendering algorithm attempts to compensate for the difference in the distances between the measured HRTF (1.9 m) and the virtual source position. This correction may not be able to correctly reproduce all cues for the evaluated range. Finally, the virtual environment was entirely anechoic, in contrast to the *real sound* condition where, despite acoustic treatment, some acoustic reflections would still exist and could be interpreted by the auditory system.

## 5. General discussion

This study presents the results of two experiments concerning localization and pointing accuracy in the peripersonal space. In contrast to numerous previous studies which have investigated auditory localization in the far-field by examining azimuth and elevation accuracy, the current studies considers near-field auditory localization associated with typical object positions, specifically for positions located in the region of a tabletop surface.

Evaluation of localization and pointing accuracy to real acoustic sources and consideration of dominant or secondary hand for the reporting task were carried out. Results showed no difference reported azimuth or distance as a function of reporting hand. Mean azimuth errors were 6.7° for frontal source positions, increasing to 17.8° for lateral positions, which were consistently underestimated (reported positions of lateral sources were shifted toward the front of the platform). These results are in contrast to a previous study by Brungart et al. (1999) which considered a similar task. However, several major differences exist between these two studies, including the reporting method (finger vs. stick pointing), source elevations, which spanned from −65° to −37° in the current study compared to −40° to 60° in Brungart et al. (1999), and acoustic conditions (the present study was conducted in a low reverberant space and not an anechoic chamber which may had influence localization in azimuth (Ihlefeld and Shinn-Cunningham, 2011).

Reported distances showed a consistent compression of reported distance toward the center of the experimental platform. Similar trends of response compression have been frequently observed in perceptual scaling paradigms that depend on the range of the presented stimuli (Parducci, 1963) as well as the setup used to collect subjects responses. For example, Zahorik (2002) observed a general overestimation of the nearest distances and an underestimation of farther distances, with distances spanning from 0.3 to 13.79 m.

Comparison of localization and pointing accuracy to real acoustic sources and visual sources of comparable duration using the same reporting technique and experimental platform showed only minor errors in the visual condition. The lack of a common bias in results between stimulus modalities indicates that the observed errors in performance are due to other factors than biomechanical difficulties in the reporting task. Mean reported azimuth errors were comparable between these two conditions. Some distance compression was observed for visual stimuli with compression being directed toward the farthest distance, while a greater degree of compression was observed for auditory stimuli where compression was directed toward the center of the middle of the platform.

A final comparison between real acoustic sources and binaurally rendered acoustic virtual sources highlighted several limitations of the binaural rendering. Reported source azimuths exhibited increased errors with azimuths being consistently overestimated toward more lateral positions. In addition, no differences were observed in reported distances relative to the rendered distance, meaning that there was no perceived distance variation between virtual sources. Numerous factors can be considered in trying to determine the cause of such lack of perception, such as the purely anechoic synthesis conditions vs. the present, while minimal, room effect of the experimental room and the use of non-individual HRTF's (despite efforts to individualize the measured dataset and the inclusion of a learning phase). In the context of an auditory guidance system in the peripersonal space, considering the observed limitations, additional cues would be necessary to aid the user in estimating the distance to the auditory target object. First, the use of a continuous sound allowing the user to move their head during localization, thus taking advantages of dynamic changes of the acoustic cues, is well known to improve directional localization (see Middlebrooks and Green, 1991). Second, Boyer et al. (2013) have highlighted the role of the auditory-motor loop in pointing to an auditory source by displaying the source position in a hand centered coordinate system. With such a shift of coordinates, the localization cue differences are largely increased when the user moves his or her hand toward the target, thus increasing movement accuracy. Finally, localization performances can be enhanced by simulating a reverberant environment (see Shinn-Cunningham et al., 2005; Kopčo and Shinn-Cunningham, 2011), by increasing the cue variations in a specific range (see Shinn-Cunningham et al., 1998), by adding continuous modification of the stimuli using a variety of sonification metaphors (see Parseihian et al., 2012), or with static and coded cues according to distance intervals using a hierarchical auditory icon system (see Parseihian and Katz, 2012a).

## Funding

This work was supported in part by the French National Research Agency (ANR) through the TecSan program (project NAVIG ANR-08TECS-011) and the Midi-Pyrénées region through the APRRTT program. Additional funding was provided by an internal research grant by the LIMSI-CNRS and the French Inter-ministerial R&D fund (project FUI-AAP14 BiLi, www.bili-project.org) concerning binaural listening with support from “Cap Digital—Paris Region.”

### Conflict of interest statement

The authors declare that the research was conducted in the absence of any commercial or financial relationships that could be construed as a potential conflict of interest.
